# A Human In Vitro Model to Study Adenoviral Receptors and Virus Cell Interactions

**DOI:** 10.3390/cells11050841

**Published:** 2022-03-01

**Authors:** Raphael L. Tsoukas, Wolfram Volkwein, Jian Gao, Maren Schiwon, Nora Bahlmann, Thomas Dittmar, Claudia Hagedorn, Eric Ehrke-Schulz, Wenli Zhang, Armin Baiker, Anja Ehrhardt

**Affiliations:** 1 Virology and Microbiology, Center for Biomedical Education and Research (ZBAF), Witten/Herdecke University, 58453 Witten, Germany; raphael.tsoukas@uni-wh.de (R.L.T.); lumenhj1937@163.com (J.G.); m.schiwon@gmx.de (M.S.); nora.bahlmann@uni-wh.de (N.B.); eric.ehrke-schulz@uni-wh.de (E.E.-S.); wenli.zhang@uni-wh.de (W.Z.); 2 Department of Anesthesiology and Intensive Care Medicine, Medical Faculty, University Hospital Cologne, University of Cologne, 50931 Cologne, Germany; 3 Bavarian Health and Food Safety Authority (LGL), 85764 Oberschleissheim, Germany; wolfram.volkwein@lgl.bayern.de (W.V.); armin.baiker@lgl.bayern.de (A.B.); 4 Institute of Immunology, Center for Biomedical Education and Research (ZBAF), Department of Human Medicine, Faculty of Health, Witten/Herdecke University, 58455 Witten, Germany; thomas.dittmar@uni-wh.de; 5 Center for Biomedical Education and Research (ZBAF), Witten/Herdecke University (UW/H), 58448 Witten, Germany; claudia.hagedorn@uni-wh.de

**Keywords:** adenovirus, receptor, CD46, CAR, knockout cell line, human, virus, tight-junction-knockout cell line, CD46-knockout cell line, CRISPR

## Abstract

To develop adenoviral cell- or tissue-specific gene delivery, understanding of the infection mechanisms of adenoviruses is crucial. Several adenoviral attachment proteins such as CD46, CAR and sialic acid have been identified and studied. However, most receptor studies were performed on non-human cells. Combining our reporter gene-tagged adenovirus library with an in vitro human gene knockout model, we performed a systematic analysis of receptor usage comparing different adenoviruses side-by-side. The CRISPR/Cas9 system was used to knockout CD46 and CAR in the human lung epithelial carcinoma cell line A549. Knockout cells were infected with 22 luciferase-expressing adenoviruses derived from adenovirus species B, C, D and E. HAdV-B16, -B21 and -B50 from species B1 as well as HAdV-B34 and -B35 were found to be CD46-dependent. HAdV-C5 and HAdV-E4 from species E were found to be CAR-dependent. Regarding cell entry of HAdV-B3 and -B14 and all species D viruses, both CAR and CD46 play a role, and here, other receptors or attachment structures may also be important since transductions were reduced but not completely inhibited. The established human knockout cell model enables the identification of the most applicable adenovirus types for gene therapy and to further understand adenovirus infection biology.

## 1. Introduction

More than 100 types of human adenoviruses (HAdV) have been described and classified into species A–G [1]. These non-enveloped viruses are of strong scientific interest due to their use in gene therapy and as threatening pathogens in immunocompromised patients. More recently, adenoviruses (Ads) gained increasing interest as vaccine vectors in the context of the ongoing global pandemic caused by the severe acute respiratory syndrome coronavirus 2 (SARS-CoV-2). The viral, double-stranded, linear DNA genome offers large packaging capacities to deliver foreign DNA of up to 37 kb [2]. Other advantages of Ads in gene therapy are their abilities to achieve high efficiency of transduction, high levels of transient gene expression and transduction of dividing and nondividing cells. Although wildtype Ad infections in healthy humans are mostly self-limiting, local outbreaks with severe courses and occasional lethality have been described in immunocompetent patients [3,4,5,6,7]. For immunocompromised patients, Ads represent a threat [8,9], and are associated with high morbidity and mortality in allogenic bone marrow transplant patients [10] and solid organ transplant patients [11]. The most common Ad types associated with disease worldwide are: HAdV-C1, -C2, -C5, -B3, -B7, -B21, -E4 and -F41 [12,13,14,15,16,17,18]. In immunocompromised patients, HAdV-C1, -C2, -C5, -A12, -A31, -B3, -B11, -B16, -B34 and -B35 are most frequently associated with disease [18,19,20,21]. Although there are anti-adenoviral drugs such as cidofovir available for preemptive therapy, treatment of Ad-associated diseases remains a challenge [22]. Binding of Ads to the host cell through surface proteins CD46, CAR and Desmoglein-2 is well-studied [23,24,25,26]. Additionally, CD80, CD86 and MARCO are described as binding structures for Ads [27,28]. CD46 belongs to the regulators of complement activation (RCA) protein family and was shown to be utilized by species B and D adenovirus exponents [23,29,30]. One of its important functions is to protect healthy cells from complement-mediated degradation. CD46 is expressed by all nucleated cells in humans. Ligand binding induces CD46 oligomerization and triggers macropinocytosis and downregulation of CD46, thereby increasing immune suppression and complement-mediated lysis [31]. Ad binding to CD46 induces an endocytic pathway, and likewise antigen presentation and cell migration in non-polarized cells [32]. CAR has been shown to bind fibers from exponents of adenovirus species A and C–E [33]. It belongs to the immunoglobulin superfamily and forms homodimers in epithelial cell–cell adhesions. CAR is expressed basolateraly on epithelial cells and is thought to be exploited by viruses for cell entry and virus release. Once the infected cells produce viral particles, they bind to CAR and open tight junctions, permitting viral escape across epithelial borders [34]. Although these proteins are described as receptors for certain Ad types, a side-by-side comparison of receptor usage by the plethora of Ad types is lacking. Comprehensively studied viruses for receptor usage are HAdV-C5 and HAdV-B35, utilizing CAR and CD46, respectively. Receptor usage is important for virus tropism due to the varying receptor expression by different cells and tissues [30,35]. For example, CAR is downregulated in many cancer cells. CAR utilizing HAdV-C5 exhibits strong liver tropism in vivo [36]. In accordance, adenoviral oncolytic vectors equipped with HAd-B35-derived fibers have been shown to transduce liver metastasis in vivo in a CD46 affinity-dependent manner [37]. On the other hand, CD46 affinity vectors will always exhibit low tissue specificity due to the nearly ubiquitous CD46 expression in humans. Recently, two concepts for modulation of adenoviral vector tropism were shown. The generation of chimeric vectors comprising fibers from different virus types was explored, and shielding techniques to avoid unintended binding to host structures were investigated [38]. A major challenge for systemic gene therapy lies in redirecting tropism of current vectors based on receptor usage. Additionally, targeted anti-adenoviral therapy could be improved by a detailed understanding of attachment mechanisms of the different adenoviral types. However, for most of the more than 100 adenovirus types, receptor usage is still unclear. Current methods do not allow adenoviral receptor analysis in a high-throughput screening manner. Recent microbiological receptor studies were based on laborious blocking assays or transient knockdown through siRNA experiments, bearing limitations due to the considerable effort required.

Most receptor studies are based on non-human CHO cells with limited transferability to the human organism. Establishing a stable human receptor knockout cell line would overcome such limitations and afford the possibility to broadly analyze receptor usage on human cells. Applying the CRISPR/CAS technology [39], we implemented a stable human knockout in vitro model, which enables side-by-side comparison of CD46 and CAR usage in Ads. By combining our GFP/luciferase tagged adenovirus library including all adenovirus species [40] with our novel human gene knockout model, we investigated AdV receptor usage in a systematic approach. To demonstrate the applicability of the model, we analyzed well-studied viruses and three of the most recently described Ads (HAdV-D70, HAdV-D73 and HAdV-D74) isolated from immunocompromised patients [41].

## 2. Materials and Methods

### 2.1. Cells and Cell Culture

Prior to the experiments, A549 cells were tested for genetic characteristics using PCR single-locus technology for authentication. Twenty-one independent PCR systems: Amelogenin, D3S1358, D1S1656, D6S1043, D13S317, Penta E, D16S539, D18S51, D2S1338, CSF1PO, Penta D, TH01, vWA, D21S11, D7S820, D5S818, TPOX, D8S1179, D12S391, D19S433 and FGA, were investigated (Promega, Walldorf, Germany, PowerPlex 21 PCR Kit). In parallel, positive and negative controls were tested, yielding correct results. Cells were certified as the A549 human lung carcinoma cell line detected in the Leibniz Institute DSMZ, German Collection of Microorganisms and Cell Cultures online database (Eurofins Medigenomix Forensik GmbH, Ebersberg, Germany). The A549 cell line is parental to all three established knockout cell lines: CAR-KO-, CD46-KO- and CC-KO-A549. All four cell lines were maintained in Dulbecco’s Modified Eagle’s Medium (DMEM) (PAN Biotech, Aidenbach, Germany), supplemented with 10% fetal bovine serum (FBS, GE Healthcare, Münster, Germany) and 100 µg/mL penicillin/streptomycin (Pen/Strep, PAN Biotech). Mycoplasma contamination testing was carried out by PCR using the mycoplasma detection Kit Venor^®^GeM Classic (Minerva Biolabs GmbH, Berlin, Germany). All cell lines tested negative for mycoplasma using the mycoplasma detection Kit Venor^®^GeM Classic (Minerva Biolabs GmbH, Germany).

### 2.2. Viruses

Wildtype viruses HAdV-D70, HAdV-D73 and HAdV-D74 were kindly supplied by Albert Heim (Deutsches Zentrum Infektionsforschung, Hannover and Braunschweig, Germany) [42,43]. HAdV-D70 was initially isolated from diarrheal feces of an immunocompromised, allogenic, hematopoietic stem cell transplant (SCT) recipient. The comparison with other HAdV-D genomes showed substantial sequence divergence, and solely the fiber gene was shown to be related with HAdV-D29, with almost 100% sequence identity [43]. HAdV-D73 was isolated from diarrheal feces of a lymphoma patient treated with chemotherapy and HAdV-D74 was initially isolated from diarrheal feces of a patient treated with allogeneic hematopoietic SCT for myelodysplastic syndrome. To analyze viral receptor usage, we directly cloned the complete genomes of the three viruses from the clinical isolates and modified them into first-generation vectors by replacing their E3 gene with an eGFP and a luciferase reporter gene, as described recently [41]. The first-generation vector p15A-HAdV17GFP with E1 gene deletion replaced by eGFP was generated by modifying p15A-HAdV17 through linear-circular homologous recombineering (LCHR) based on ccdB selection. Other luciferase-tagged viruses were generated as described earlier [40].

### 2.3. Customization of CRISPR/Cas9 Plasmid

For transfection of A549 cells, we customized the plasmid pShV-CBh-Cas9 as described in [44]. For the CD46 knockout, we separately inserted three different gRNAs into the pShV-CBh-Cas9 plasmid. All three gRNAs represent binding sides on different exons of the CD46 gene. The respective exons are essential for the expression of all 14 subtypes of CD46. The following oligonucleotides were inserted: gRNA1: 5′-CAC CGC GTT CAC CAA TCT CAT AGT A-3′, gRNA2: 5′-aaactatatacgggatcctttaaac-3′ and gRNA3: 5′-CAC CGC AAT TGT GTC GCT GCC ATC G-3′. The resulting customized plasmids were named: pShV-CBh-Cas9-gRNACD46-1, pShV-CBh-Cas9-gRNACD46-2 and pShV-CBh-Cas9 -gRNACD46-3. The gRNA sequences we selected using the web-based service of CRISPOR.tefor.de. Sequences met the following criteria: (a) high out of frame score and (b) lowest number of off-target sites. The same procedures were applied for the plasmid customization of the CAR knockout. Here, the following three oligonucleotides were inserted into the plasmid: gRNA1: 5′-CAC CGC GTG CTC CTG TGC GGA GTA G-3′, gRNA2: 5′-CAC CGG CTT TTT CAA TCA TCT CTT C-3′ and gRNA3: 5′-CAC CGC GTA AAT TTG CAT GGC AGA T-3′. These three gRNAs represent binding sites on two different exons essential for the expression of all CAR subtypes derived from alternative splicing.

### 2.4. Transfection of A549 Cells

A549 cells were plasmid-transfected using Lipofectamin^®^ LTX and PLUS™ Reagent (Invitrogen ™). Cells of 80% confluency were transfected with 2.5 µg of plasmid-DNA, diluted in 500 µL of Opti-MEM I (Gibco™), 2.5 µL of PLUS™ Reagent and 10 µL of Lipofectamin in a 6-well format. After 24 h of incubation at 37 °C and 5% CO_2_, the transfection mix was exchanged with growth medium. Cells were then either cultured for further use or DNA was isolated 72 h after transfection.

### 2.5. Sorting of Knockout Cells and Cloning

On day 10 post-transfection, cells were sorted by magnetic cell sorting (MACS). Therefore, 2 × 10^6^ cells diluted in 1 mL of PBS were incubated with 2 µg of the primary antibody for 10 min at 4 °C. For sorting CD46 knockout cells, we used monoclonal mouse anti-human CD46 antibody, clone MEM-258 (GeneTex Inc., Irvine, CA, USA). For sorting CAR knockout cells, the monoclonal CAR antibody CAR (E1-1) sc-56892 (Santa Cruz Biotechnology Inc., Dallas, TX, USA) was used. Cells were washed twice with PBS supplemented with 0.1% BSA and 2 mM of EDTA and resuspended in 1 mL of PBS. One hundred µL of Dynabeads^®^ Pan Mouse IgG (Thermo Fisher Scientific, Waltham, MA, USA) was added and incubated for 30 min at 4 °C by continuous tilting and rotating. The tubes were then placed on a magnet for 2 min. Cells in the supernatant were transferred to a fresh tube for further culturing, while the cell pellet collected on the magnet was discharged. Thereby, negative cells not expressing the respective surface structure (CD46 or CAR) were separated from positive cells. Magnetic cell sorting was repeated successively several times. For CAR knockout cells, magnetic cell sorting was inefficient, and thus sorting by flow cytometry (FACS) was performed. Here, 2 × 10^6^ cells per sample were washed twice with PBS and diluted in PBS, followed by incubation with the primary antibody CAR (E1-1) sc-56892 (Santa Cruz Biotechnology Inc. USA) for 30 min at 4 °C. After washing twice with PBS, cells were incubated with the fluorophore-tagged secondary antibody goat anti-mouse IgG, F(ab′)_2_-APC: sc-3818 (Santa Cruz Biotechnology Inc. USA). Cells were washed again and sorted using the FACSCalibur (Becton Dickenson, Heidelberg, Germany). Sorted cells were cultured for four passages, followed by flow cytometry analysis for CAR or CD46 expression. Populations exhibiting 70% of receptor-negative cells were singularized. Therefore, a serial dilution of cells was seeded in 96-well plates and single-cell clones were picked.

### 2.6. Flow Cytometry Analysis

Flow cytometry analysis was used to assess the receptor expression of knockout cells and normal A549 cells, to measure the sorting efficiency in the process of knockout cell production. To assess receptor expression and to identify knockout clones, cells were stained with the following monoclonal antibodies directed to the human surface molecules: CD46 antibody, clone MEM-258 (GeneTex Inc. USA), and from Santa Cruz Biotechnology Inc., USA: CAR (E1-1) sc-56892, CD80 B7-1 (F-7): sc-376012, CD86 B7-2 (D-6): sc-28347 and dsg2 (AH12.2): sc-80663. Then, 1 × 10^5^ cells were washed twice with 1% FBS in PBS, followed by incubation with the respective antibodies. After incubation with the APC-linked secondary antibody (goat anti-mouse IgG, F(ab´)_2_-APC: sc-3818, Santa Cruz Biotechnology Inc., USA), samples were washed two times. Flow cytometric analysis was performed using the Gallios (Beckmann Coulter^®^, Brea, CA, USA) and Software Version: Gallios 1.2 (Beckmann Coulter^®^). The median of fluorescence intensity (MFI) was evaluated for triplicates. Viral transduction was measured as the MFI.

### 2.7. Isolation of Genomic DNA

Genomic DNA from A549, A549-KO-CD46, A549-KO-CAR and A549-KO-CD46/CAR cells was isolated using the QIAamp DNA Blood Mini Kit (QIAGEN GmbH Hilden, Germany) according to the manufacturer’s instructions.

### 2.8. T7 Endonuclease I (T7EI) Mutation Detection Assay

To analyze the genomic region of CD46, a 609 bp region spanning the CRISPR/Cas target site was amplified from isolated genomic DNA using the primer pair T7EI CD46 fw (AAT GAC AGC CAG CCA AGT TTT C) and T7EI CD46 rev (TTC GTG TCA TTC ATC TGG CA). The same approach was used for the CAR region. Here, an 829 bp region spanning the CRISPR/Cas target site was amplified using the primer pair T7EI CAR fw (CTT AAA GGA GGG CGC CAA CG) and T7EI CAR rev (AGC AAA GAA AGG CAA GCC CG). All oligonucleotides were synthesized by Integrated DNA Technologies (IDT, Leuven, Belgium). The resulting PCR products were then subjected to T7EI mutation detection analysis using the EnGen^®^ Mutation Detection Kit (New England Biolabs, Frankfurt am Main, Germany) according to the manufacturer’s instructions. The resulting digests were analyzed using the CRISPR Discovery Gel Kit (Agilent, Santa Clara, CA, USA) in combination with a 5200 Fragment Analyzer System (Agilent) according to the manufacturer’s instructions.

### 2.9. Analyzing Transduction Efficiencies In Vitro

For the luciferase assay, 30 × 10^3^ cells/well were seeded in 96-well plates. After 4 h of incubation, the growth medium was discharged from adherent cells and replaced with luciferase-tagged virus diluted in fresh medium. We used 2, 8 and 20 physical viral particles per cell. Twenty-six hours post-infection, reconstituted Nano-Glo^®^ Luciferase Assay Reagent (Promega, Walldorf, Germany) was added to the cells. After 5 min of incubation at room temperature, the luminescence of the cell lysate was measured in a TECAN^®^ Platereader. For the measurement of viral transduction by flow cytometry, 2.5 × 10^4^ cells were seeded in 24-well plates. After 4 h of incubation, the medium was replaced with eGFP-tagged virus diluted in growth medium. Since the expression of eGFP is lower than luciferase expression efficiency in our viruses, here, we used 1 × 10^3^ physical viral particles per cell. Forty-eight hours post-infection, cells were washed with PBS and detached with trypsin-EDTA. We resuspended cells in Dulbecco’s minimal essential medium (DMEM) containing 10% FBS, centrifuged, washed with PBS and fixated cells with 4% formaldehyde. Fluorescence was measured by analyzing 5000 single cells using a Gallios (Beckmann Coulter ^®^) flow cytometer and Beckmann software. Samples were excited with 488 nm and signals were detected using the FITC channel (525/40 nm). The background signal was obtained by analyzing uninfected cells (negative controls). The percentage of GFP-expressing cells was determined by selecting the region of fluorescence above the background level of auto-fluorescence from uninfected cells.

## 3. Results

### 3.1. Receptor Expression Characteristics in A549 Cells

**Figure 1:**
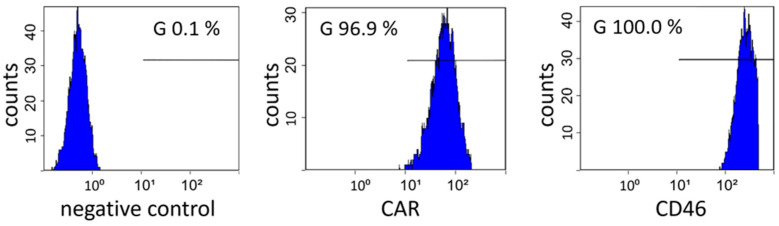
Expression of surface proteins involved in adenoviral cell entry on A549 cells. For CD46, we measured 100% positive cells, and for CAR, 96.4% positive cells. The negative control was analyzed without the use of a primary antibody.

A549 cells are described as adenocarcinomic human alveolar basal epithelial cells, which were established from cancerous lung tissue [45]. They are widely used as a lung epithelial cell model and as a host cell line of many respiratory viruses, including adenoviruses. This prompted us to modify this human cell line in order to study adenoviral receptor usage. To characterize these cells, we measured the expression levels of cell surface proteins described as adenoviral attachment molecules or receptors. FACS analysis revealed that A549 cells express CD46 at high levels and CAR at lower levels (Figure 1). Other surface molecules important for cell entry of adenoviruses are DSG2, CD80 and CD86 [46,47]. For DSG2, we detected a positive signal for 98.6% of the analyzed cells, and for CD80 and CD86, no expression was detected on A549 cells (Supplementary Table S1).

### 3.2. Construction and Effectivity of the Customized CRISPR/Cas9 Plasmids

**Figure 2:**
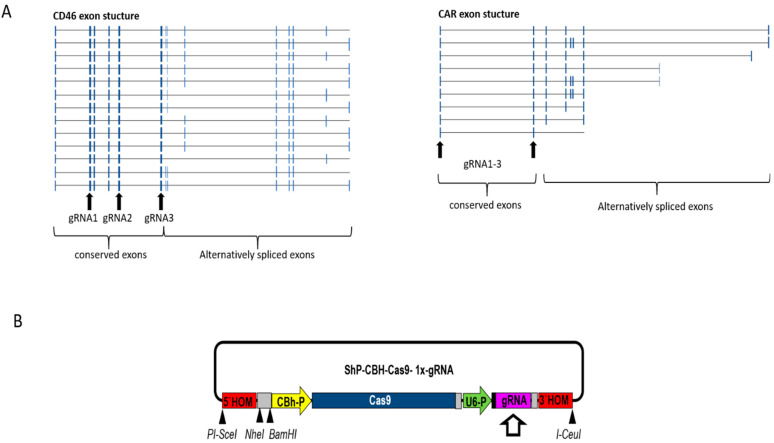
Exon structure of the CD46 and CAR genes and the CRISPR/Cas9 plasmid used for generation of the knockout cell lines. (**A**) Arrow-marked exons in the CD46 and CAR gene regions containing the selected target sites for the gRNAs are translated in all protein isotypes. (**B**) Insertion of gRNA into the customized shuttle vector pShV-CBh-Cas9.

We applied the CRSIPR/Cas9 system to generate stable human knockout cell lines for CD46 and CAR. Both CD46 and CAR exhibit varying degrees of alternative mRNA splicing, creating multiple protein isoforms [46]. We aimed at knocking out all isoforms of CD46 and CAR. Applicable target sites in early exons were identified in the CD46 and CAR genes essential for all isoforms of the two receptors. Appropriate target sites were filtered to minimize off-target activities and selected for the high binding affinity of gRNA. For each receptor, we identified three possible target sites, from which we used one in the final knockout approach. Target sites for CD46 were located on exons 2, 5 and 6 and target sites for CAR were located on exons 1 and 2 (Figure 2A). These exons are essential for the expression of all protein isoforms, and NHEJ-mediated frameshifts within these exons would terminate the translation of all protein isoforms. Finally, the gRNAs consisting of 25 bp were separately inserted to the pShV-CBh-Cas9 plasmid (Figure 2B).

### 3.3. Generation of CD46 and CAR Knockout Cell Lines

**Figure 3:**
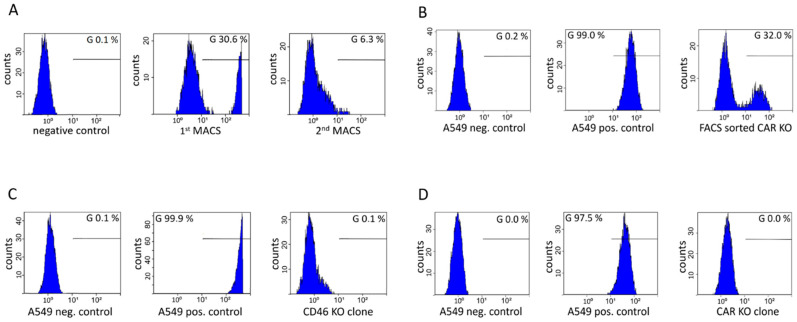
Generation of CD46 and CAR knockout cell lines using cell sorting. (**A**) CD46 protein expression after the first and second round of the MACS procedure. (**B**) CD46 protein expression after cell singularization of the CD46 knockout cells. The chosen clone CD46-KO is displayed. (**C**) CAR protein expression after flow cytometric sorting and (**D**) after cell singularization. The chosen CAR knockout clone, CAR-KO, is displayed. The parental A549 cell line serves as a positive control, incubated with the corresponding primary and secondary antibodies. For the negative control, the parental A549 cells were incubated with the secondary antibody only. All measurements were carried out with the same monoclonal APC-linked secondary antibody.

We co-transfected A549 cells with gRNA-equipped pShV-CBh-Cas9 and a plasmid encoding for eGFP expression to monitor transfection efficiency. To separate successful knockouts from unmodified cells, we performed magnetic cell sorting (MACS) using a primary antibody specific for human CD46. After the expansion of sorted cells and consecutive magnetic sorting, a subsequent FACS analysis revealed a substantial reduction in CD46-positive cells (Figure 3A). Subsequently, cells were seeded as individual clones in 96-well plates and expanded. Six clones were analyzed by FACS (Supplementary Figure S1) and one clone was selected for further analysis and culture, because this clone (CD46-KO) showed complete removal of CD46 positivity (Figure 3B).

For the generation of a CAR knockout cell line, we used the same approach as described for CD46. In addition, after repeated MACS, we sorted cells by FACS, yielding a population with 70% CAR-negative cells (Figure 3C). After singularization in a 96-well plate and expansion of 8 clones, these clones were analyzed by FACS for CAR expression (Supplementary Figure S2). One clone (CAR-KO) which showed complete absence of CAR protein expression was selected for further analysis and culture (Figure 3D).

### 3.4. Generation of a CD46/CAR Double Knockout Cell Line

**Figure 4:**
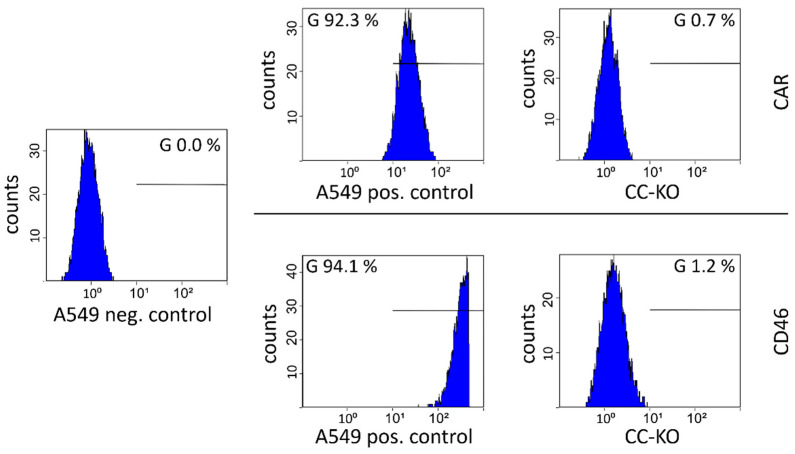
Establishment of a CD46/CAR double knockout cell line. The selected clone CC-KO exhibits a loss of CAR and CD46 protein expression in the FACS measurement. As the positive control, the parental A549 cell line incubated with the same primary antibodies was used. For the negative control, the parental A549 cells were incubated with the secondary antibody only. All measurements were carried out with the same monoclonal APC-linked secondary antibody.

To investigate adenoviral receptors, we strived to generate a human cell line lacking both important surface proteins CD46 and CAR. Therefore, the successfully cloned CD46 knockout cell line was transfected with the respective CRISPR/Cas9 plasmid-expressing gRNA for the CAR target site. Further steps were similar to the generation of the single CAR knockout cell line described above. Sixteen clones were analyzed for CAR expression (Supplementary Figure S3). Clone 14 exhibited a complete loss of CD46 and CAR expression via the FACS analysis and was selected for further analysis and culture. Results from the FACS analysis are depicted in Figure 4, showing the removal of CD46 and CAR expression on the double knockout cell line (CC-KO) compared to the parental A549 cell line. This measurement was repeated more than three times over ten passages to assure the stability of the phenotype concerning the receptor expression. The knockout was stable over passages without the use of selection substances. The knockout cell lines were cultivated under the same conditions as the parental A549 cells.

### 3.5. Genetic Characterization of Knockout Cell Lines

**Figure 5:**
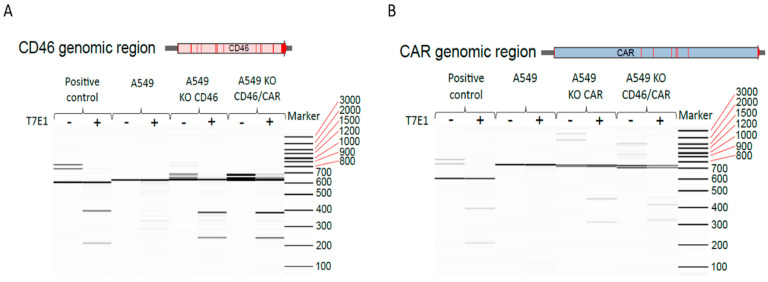
Analysis of the genome-edited CD46 and CAR loci in A549 cells. (**A**) To further confirm genome editing at the CD46 target locus, a T7 endonuclease I (T7EI) assay was performed. For this purpose, the genomic region of CD46 spanning the CRISPR/Cas target site was amplified by PCR and analyzed by the T7EI assay. Results are shown for the CD46 genomic region in A549, A549 KO CD46 and A549 KO CD46/CAR cells. Cleavage of the PCR product resulting in two smaller fragments indicates a CRISPR/Cas edit within the generated PCR product. The positive control was derived from the EnGen^®^ Mutation Detection Kit (New England Biolabs, MA, USA) and the PCR products were either T7EI-digested (+) or not (−). (**B**) To further confirm genome editing at the CAR target locus, a T7 endonuclease I (T7EI) assay was performed. For this purpose, the genomic region of CAR spanning the CRISPR/Cas target site was PCR-amplified. Cleavage of the PCR product resulting in two smaller fragments indicates a CRISPR/Cas edit within the generated PCR product. Results are shown for the CD46 genomic region in A549, A549 KO CD46 and A549 KO CD46/CAR cells. The positive control derived from the EnGen^®^ Mutation Detection Kit (New England Biolabs, MA, USA) and the PCR products were either T7EI-digested (+) or not (−).

To confirm genome editing at the CD46 and CAR target loci introduced by the CRISPR/Cas9 machinery, a T7 endonuclease I (T7EI) assay was performed. We observed a slight shift of the PCR products compared to the PCR products of the parental cell line in capillary gel electrophoresis. The results of the T7EI assay for the CD46 gene region of the single CD46 and CD46/CAR double knockout cell lines are depicted in Figure 5A. Results for the CAR gene region hinting towards different deletions in single CAR and double CD46/CAR knockout cell lines are depicted in Figure 5B.

### 3.6. CD46 and CAR Receptor Usage of Adenovirus Types from Species B to E

**Figure 6:**
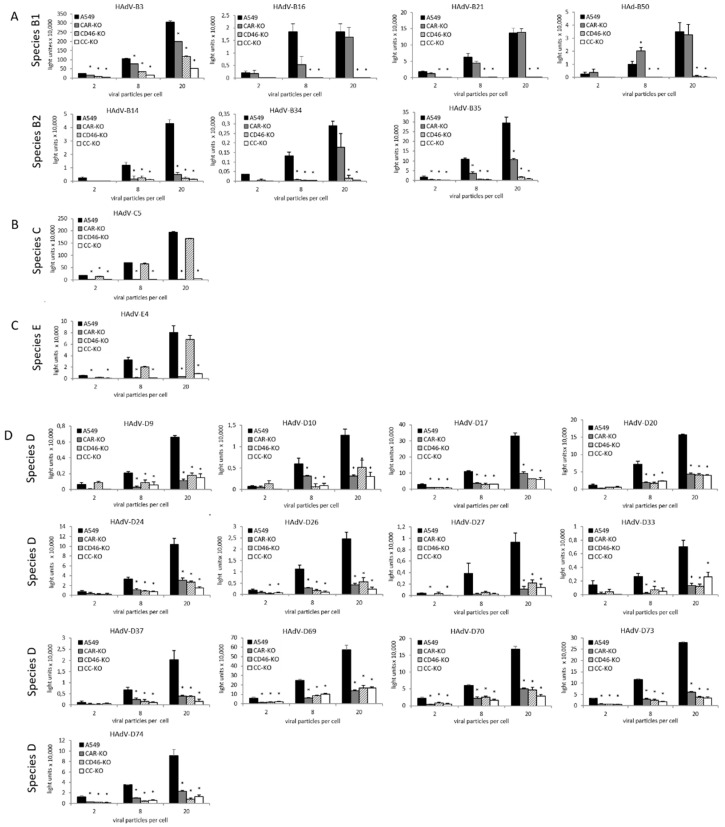
Screening of receptor usage in adenovirus species B, C and E adenoviruses. Parental A549, CAR-KO, CD46-KO and CC-KO cells were transduced with respective viruses at varying viral particle numbers per cell (VP/C: 2, 8, 20). Luminescence levels were analyzed 26 h post-infection. Uninfected cells were used for the background level. Each bar represents the mean of triplicated wells (96-well plate). Error bars represent standard deviation. Each of the KO cell lines was compared to the A549 cell line. Significant luminescence reductions are marked by * (*p* < 0.05). Each graph represents data from one out of three independent experiments. All experiments are depicted in Supplementary Figure S4.

To investigate the receptor usage of adenoviruses from different species, we screened viruses from our virus library engineered and equipped with a luciferase reporter gene [40]. Transduction efficiencies in the four cell lines: A549 (parental), CD46-KO, CAR-KO and CC-KO, were compared for each of the viral vectors by a luciferase assay. Cells were infected at 2, 8 and 20 viral particles per cell. A significant reduction of viral uptake in one of the single knockout cells was categorized as a receptor using (+). A reduction of viral uptake to less than 1% of the uptake in the A549 parental cells was categorized as receptor-dependent (++). If a virus in all three experiments performed showed a tendency for using a receptor with no significance, we categorized this as receptor usage not excluded (?). Table 1 provides a summary of all analyzed viruses and their receptor usage. Figure 6 shows an overview of luciferase reporter assay outcomes of species B, C, D and E viruses. Species B1 viruses HAdV-B16, -B21 and -B50 exhibited a CD46 dependency, whereas HAdV-B3 was shown to use CD46 and CAR for cell entry, although other cellular entry receptors may be involved, too (Figure 6A). Species B2 viruses HAdV-B34 and -B35 were shown to be CD46-dependent and HAdV-B14 was shown to use CD46 and CAR, but other attachment structures or receptors may also be involved (Figure 6B). Species C virus HAdV-C5 exhibits CAR dependency (Figure 6C). Species E virus was shown to be CAR-dependent (Figure 6D). All screened species D viruses: HAdV-D9, -D10, -D17, -D20, -D24, -D26, -D27, -D33, -D37, -D69, -D70, -D73 and -D74, showed usage of both CD46 and CAR but not dependency.

## 4. Discussion

Based on the human adenocarcinoma alveolar epithelial cell line A549, three stable knockout cell lines were established carrying knockouts for CAR, CD46 or a double knockout for CAR and CD46. Transduction experiments with the well-studied adenoviruses HAd-C5 and HAdV-B35 confirmed existing data indicating that these viruses use CAR and CD46, respectively, for cell entry [23,25,30]. With the new cell lines, we also investigated receptor usage of the recently discovered human adenoviruses HAdV-70, HAdV-73 and HAdV-74. Our results indicate that HAdV-D70, HAdV-D73 and HAdV-D74 use both CAR and CD46 for cell entry in vitro. This is consistent with our recently published data on these three viruses [41].

The A549 cell line serves as a common model in adenoviral research, representing a standard reference cell line. Originating from the human alveolar epithelium, it resembles a natural host with reliable infection rates by adenoviruses. However, as an immortalized cancer cell line, genetic alterations may result in modified protein expression compared to healthy alveolar epithelial cells. As a lung cancer-derived cell line, it is likely to express CAR on a higher level compared to healthy alveolar cells since lung cancers are shown to have elevated CAR expression levels [48]. This human cell model does not represent an in vitro model imitating lung tissue, nor is it meant to be a model for adenoviral tropism. We chose the A549 cell line as the parental cell line as it is phenotypically close to the healthy human cell, while at the same time providing us with the benefits of a widely used immortalized cell line, namely standardized culture conditions and reproducibility. As the in vivo infection process of adenoviruses is complex, with interactions with blood components, tissue structures hampering the direct virus cell contact and neutralizing immune response, this model cannot represent or make predictions for the in vivo infection process [49,50]. With the set of parental A549 cells and the three generated knockout cell lines, we established a reliable human in vitro model to study adenoviral receptors. The CRISPR/Cas9 engineered knockout cell lines carry a stable gene deletion for the respective receptors and do not express these proteins. Due to the knockout technique, no further selection substance is needed in culture, which could influence cell properties and the results. Parental cells and knockout cells can be cultured using the same conditions. In contrast, previous adenovirus receptor studies were reliant on complex blocking assays, for instance using fiber knob proteins in competition assays or antibodies.

A recent study described a DSG-2 and CD46 single and double knockout cell line based on A549 cells, which was used to investigate the receptor usage of human adenovirus type 55 [47]. This study revealed that this virus type mainly uses DSG-2 as an entry receptor and underlines the value of our generated cell lines. Another study describes the use of single CD46 knockout ARPE-19 cells in confirming a role of CD46 in viral entry and dissemination of CMV in this epithelial cell line [48]. This shows the successful use of human knockout models outside the adenoviral field.

In this study, we screened through our engineered virus library for CAR and CD46 receptor usage by adenoviruses from species B to E. Thereby, we identified viruses that depend on one of the two receptors for cell entry. CD46-dependent viruses were HAdV-B16, -B21 and -B50 from species B1, and HAdV-B34 and -B35 from species B2. CAR-dependent viruses were HAdV-5 from species C and HAdV-4 derived from species E. The other screened viruses, HAdV-B3 and -B14 from species B1 and B2 and all species D viruses, appear to use both CAR and CD46 for cell entry, but other receptors or attachment structures may also play a role since transductions were only reduced but not completely inhibited. For adenoviruses from species D, sialic acid (SA) has been described as a receptor. A previous review described receptor usage roughly by pooling viruses from one species [30]. This screening showed a uniform receptor usage concerning CAR and CD46 for species D viruses. On the other hand, we have recently shown that sialic acid is not used by HAdV-D17 [49] but plays a role in the cellular uptake of HAdV-D37 [49]. This indicates that viruses from the same species can have similarities in receptor usage, but especially, the viruses exhibiting no strong dependency on one surface structure should be studied for further receptor usage to gain a better understanding of tropism and virus cell interactions.

Whether CAR plays the same role for infection of these viruses in vivo is questionable since the accessibility of CAR as a tight junction protein [50] in tissue might be reduced compared to in vitro settings. Therefore, compared to CAR, CD46, exhibiting higher accessibility to viruses, could play a more prominent role in vivo. A limitation of our experiments, which we share with other receptor studies, is that the importance of other receptors, such as DSG2 and sialic acid CD80 or CD86, cannot be reflected in our data.

For the recently described adenoviruses HAdV-D70, HAdV-D73 and HAdV-D74, genetic analysis of fiber genes has shown a relation to other species D adenoviruses in BLAST analysis [43]. However, this is the first study to perform receptor assays with these viruses in a human cell line, reporting that CAR and CD46 are utilized by HAdV-D70, HAdV-D73 and HAdV-D74. Our data indicate that there may be other mechanisms of cell entry in A549 cells for these three viruses in addition to CAR and CD46, since transduction in knockout cells is reduced but not completely inhibited.

With the generated set of human knockout cell lines, we established a reliable tool to study adenoviral receptors in vitro. The knowledge of receptor usage is important for the development of new adenoviral vectors for gene therapy and is essential for the development of anti-infective drugs. Adenoviruses are a recognized threat to immunocompromised patients [8,9,10]. However, only a small fraction of adenoviruses has been investigated for receptor usage in basic virology. Here, we described CAR and CD46 receptor usage in human cells for three recently described viruses, HAd-D70, HAd-D73 and HAd-D74, which had been isolated from immunocompromised patients. We introduced a novel human knockout in vitro model for the analysis of viral receptor usage. The combination of our library comprising many reporter gene-expressing adenoviral vectors [40] with the established knockout cell lines enables broad screening of adenoviral serotypes for receptor usage in the future. Furthermore, the in vitro model can be used to measure viral uptake and thereby pave the way for studying CAR and CD46 receptor usage of other viruses and organisms. To draw a full picture of receptor usage for the broad spectrum of adenoviruses, further studies of possible receptors are needed. Insights from such studies could help to develop anti-infective therapies for threatening viruses based on cell entry mechanisms.

## Figures and Tables

**Table 1:** Receptor usage by adenoviral types from species B to E.

Species and Type	CD46	CAR
B1		
HAdV-3	+	+
HAdV-16	++	n.e.
HAdV-21	++	n.e.
HAdV-50	++	
B2		
HAdV-14	+	+
HAdV-34	++	n.e.
HAdV-35	++	n.e.
C		
HAdV-5		++
D		
HAdV-9	+	+
HAdV-10	+	+
HAdV-17	+	+
HAdV-20	+	+
HAdV-24	+	+
HAdV-26	+	+
HAdV-27	+	+
HAdV-33	+	+
HAdV-37	+	+
HAdV-69	+	+
HAdV-70	+	+
HAdV-73	+	+
HAdV-74	+	+
E		
HAdV-4	n.e.	++

++ Receptor dependency in this cell model. + Receptor usage in this cell model. n.e.: Receptor usage cannot be excluded in this cell model.

## Supplementary Materials

The following supporting information can be downloaded at: https://www.mdpi.com/article/10.3390/cells11050841/s1, Figure S1: Flow Cytometry Analysis of CD46-KO clones; Figure S2: Flow Cytometry Analysis of CAR-KO clones; Figure S3: Flow Cytometry Analysis of CD46/CAR-KO clones; Figure S4: Screening of receptor usage in adenovirus species B to E, independent expreiments; Table S1: Immunostaining of A549 cells.
