# Supplementary material for: A Human In Vitro Model to Study Adenoviral Receptors and Virus Cell Interactions
Source: Cells. 2022 Mar 1;11(5):841. doi: 10.3390/cells11050841 (PMC8909167; doi:10.3390/cells11050841)
Supplement: Supplementary file 1 [file cells-11-00841-s001.zip › cells-1556775-supplementary.pdf]

Supplementary Material

Table S1. Immunostaining of A549 cells

AdV Receptor	Percentage of Gated Cells
CD80	0.1%
CD86	0.7%
Desmoglein-2	98.6%

Expression of major adenovirus receptors on the A549 cells. To detect CAR (Coxsackievirus and adenovirus receptor) expression on the cell surface of different tumor cell lines using flow cytometry, 1×10e5 cells were washed with PBS supplemented with 1% BSA, centrifuged (500 g, 3 min), and resuspended in 100 µl PBS/BSA and 1 µl PE- conjugated rabbit anti-hCAR antibody (Antibody Online, ABIN2649016). Following an incubation step at room temperature for 1 hour cells were washed again with PBS/BSA, to remove unbound antibodies and resuspended in 100 µl PBS for flow cytometry using FACS (Beckman Coulter Gallios Flow cytometer). As controls each cell line without antibody was used. The PE-conjugated mouse anti-human CD46 antibody (Thermofisher, 12-0469-42) was used to detect surface expression of CD46 on different cell lines. For DSG-2 detection, PE-conjugated mouse anti-human Desmoglein 2 antibody was used (Thermofisher, CSTEM28).

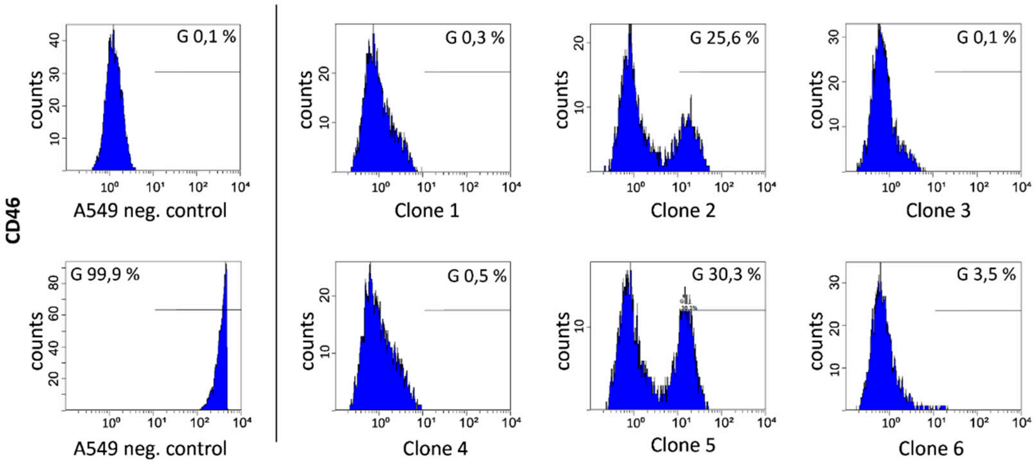

Figure S1. Flow Cytometry Analysis of CD46-KO clones. Clones 1, -3 and -4 show complete CD46 removal. Clone 3 was selected for further culture and analysis. The parental A549 cell line serves as positive control incubated with the same primary and secondary antibodies. For negative control the parental A549 cells were incubated with the secondary antibody only. All measurements were carried out with the same monoclonal APC linked secondary antibody.

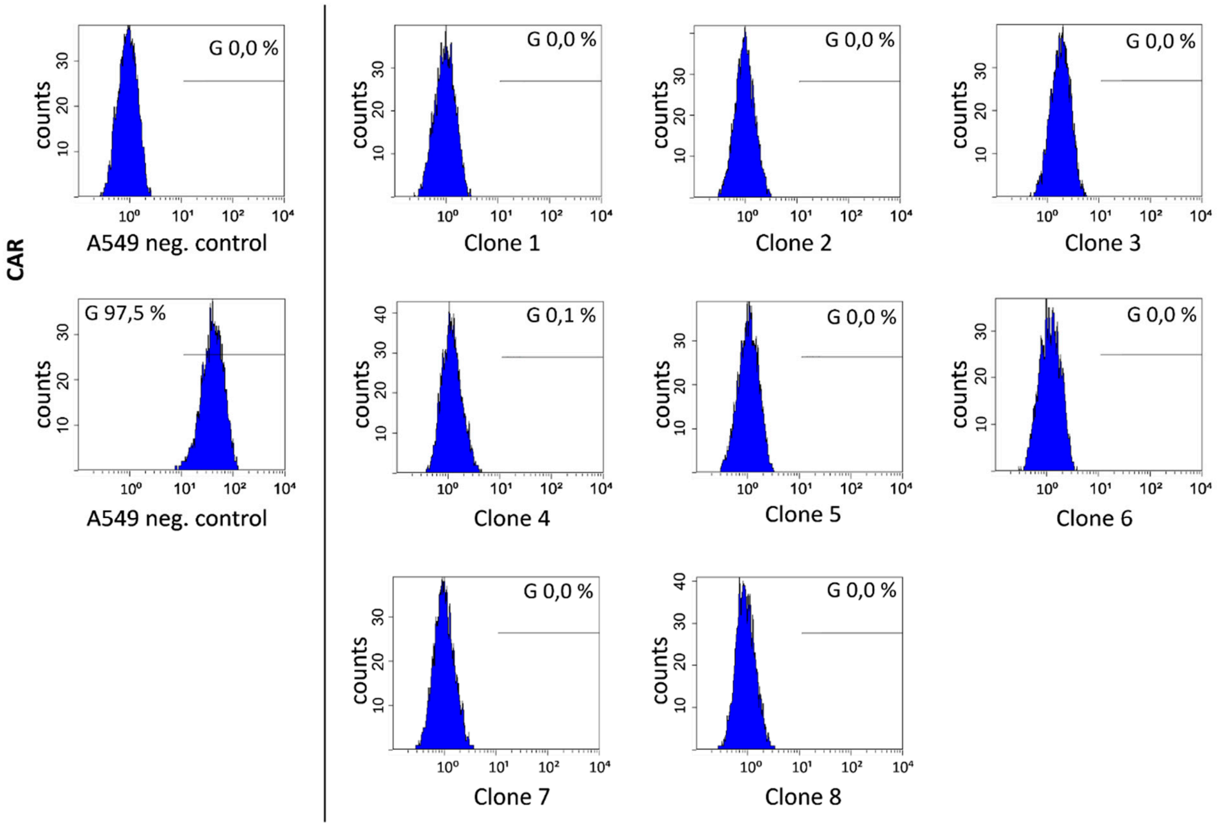

Figure S2. Flow Cytometry Analysis of CAR-KO clones. All clones showed complete CAR removal. Clone 1 was selected for further culture and analysis. The parental A549 cell line serves as positive control incubated with the same primary and secondary antibodies. For negative control the parental A549 cells were incubated with the secondary antibody only. All measurements were carried out with the same monoclonal APC linked secondary antibody.

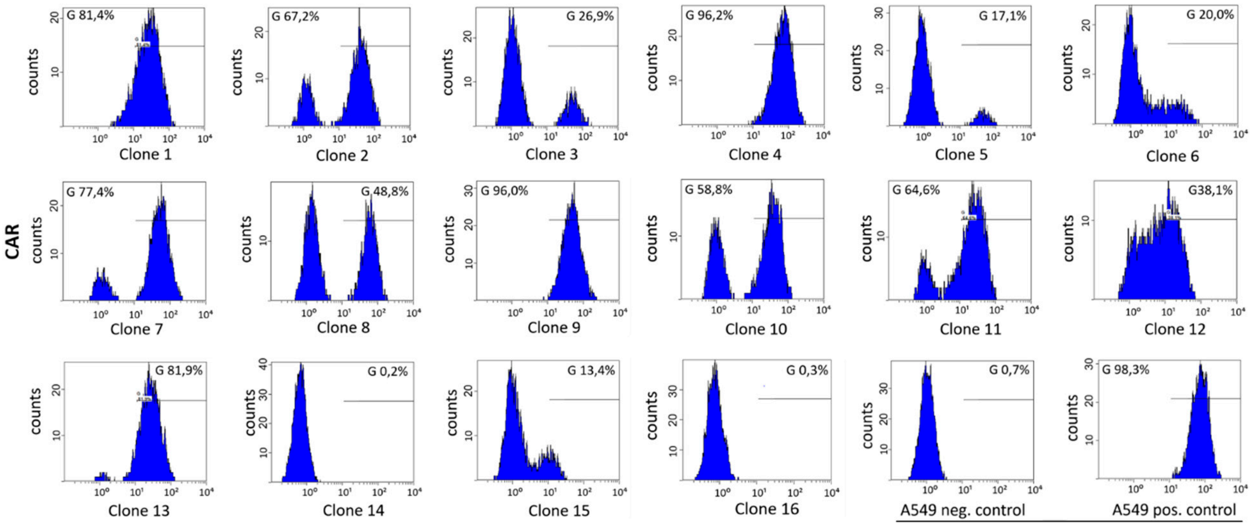

Figure S3. Flow Cytometry Analysis of CD46/CAR-KO clones. Clones 14 and 16 showed complete CAR removal. Clone 14 was selected for further culture and analysis. The parental A549 cell line serves as positive control incubated with the same primary and secondary antibodies. For negative control, the parental A549 cells were incubated with the secondary antibody only. All measurements were carried out with the same monoclonal APC linked secondary antibody.

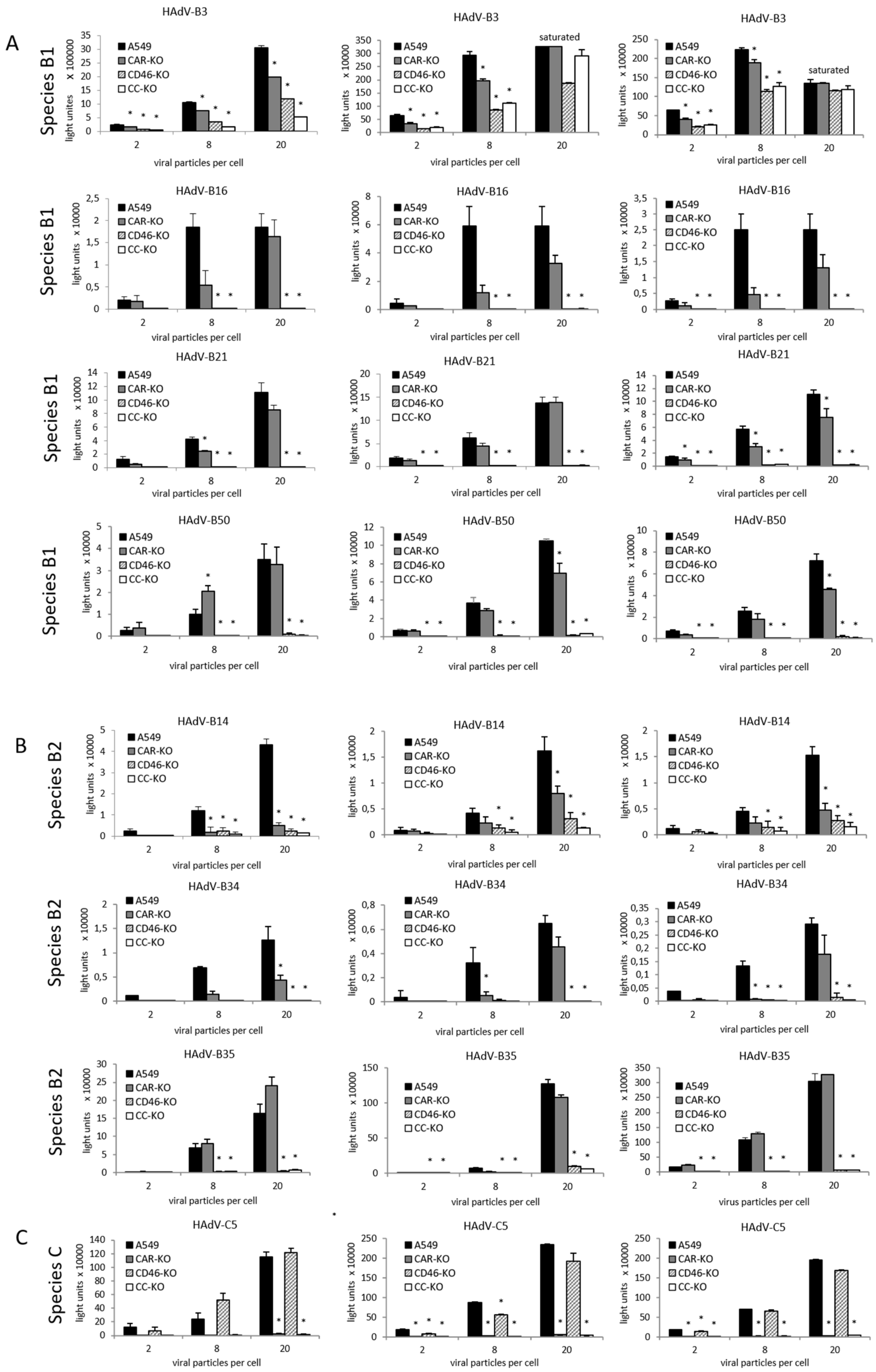
A
Species B1
HAdV-B3
saturated
HAdV-B16
HAdV-B21
HAdV-B50
A549
CAR-KO
CD46-KO
CC-KO
light unites x 100000
light units x 10000
viral particles per cell
B
Species B2
HAdV-B14
HAdV-B34
HAdV-B35
virus particles per cell
C
Species C
HAdV-C5

D

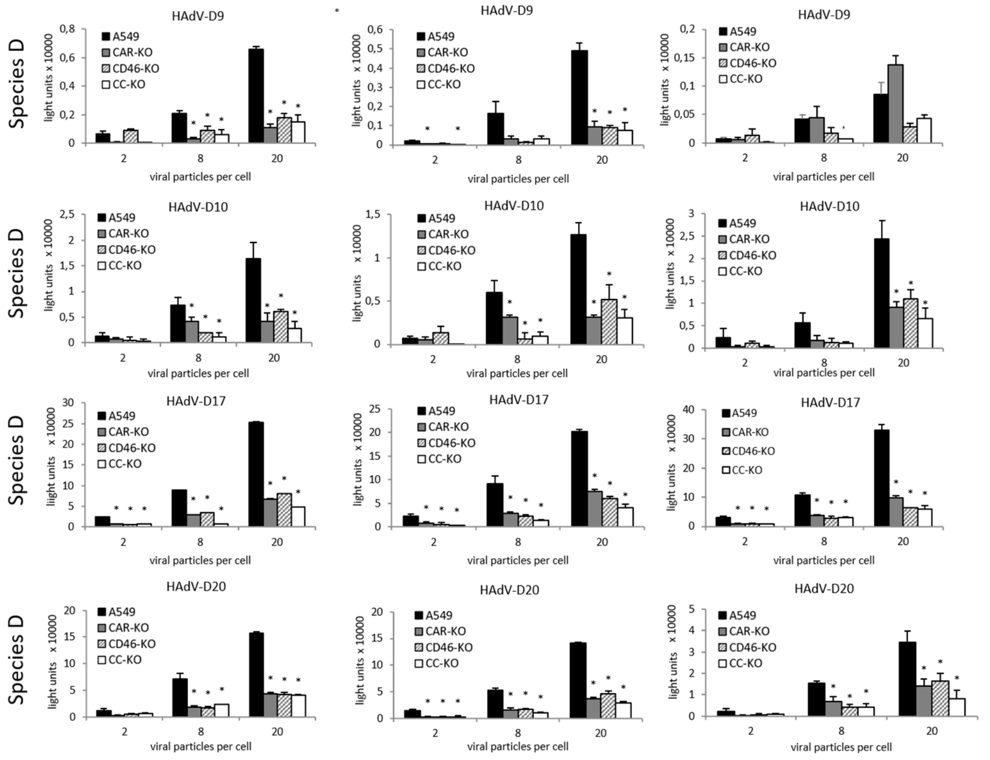
Species D
HAdV-D9
HAdV-D9
HAdV-D9
Species D
HAdV-D10
HAdV-D10
HAdV-D10
Species D
HAdV-D17
HAdV-D17
HAdV-D17
Species D
HAdV-D20
HAdV-D20
HAdV-D20
A549
CAR-KO
CD46-KO
CC-KO
light units x 10000
2
8
20
viral particles per cell

D

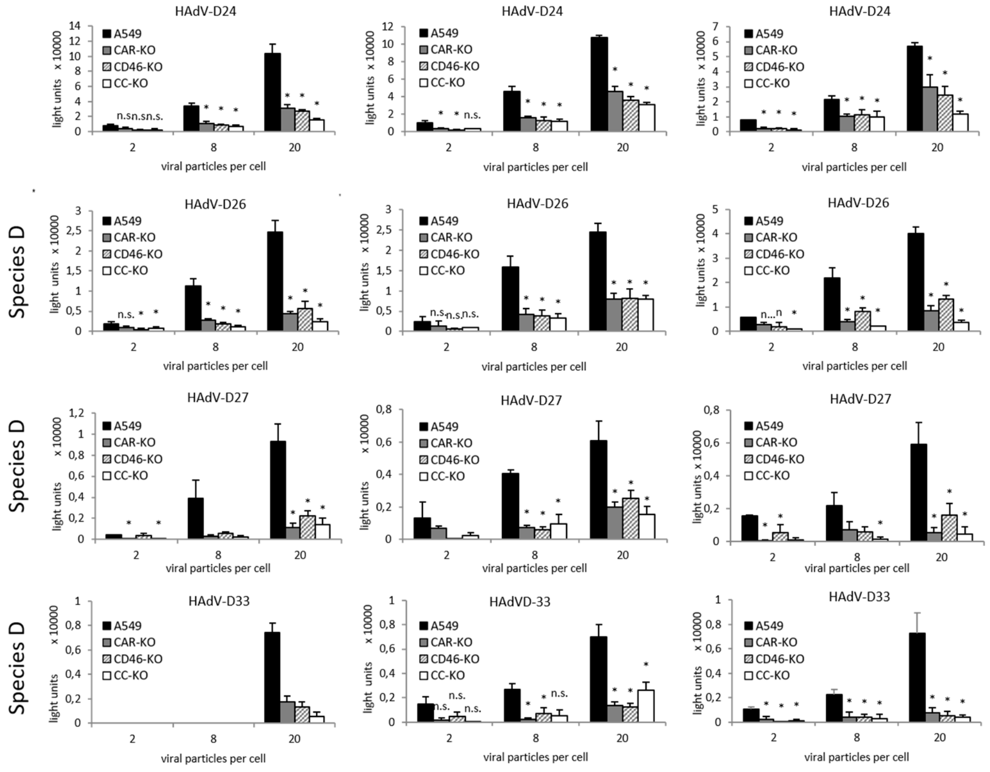
HAdV-D24
HAdV-D24
HAdV-D24
Species D
HAdV-D26
HAdV-D26
HAdV-D26
Species D
HAdV-D27
HAdV-D27
HAdV-D27
Species D
HAdV-D33
HAdVD-33
HAdV-D33
A549
CAR-KO
CD46-KO
CC-KO
light units x 10000
2
8
20
viral particles per cell

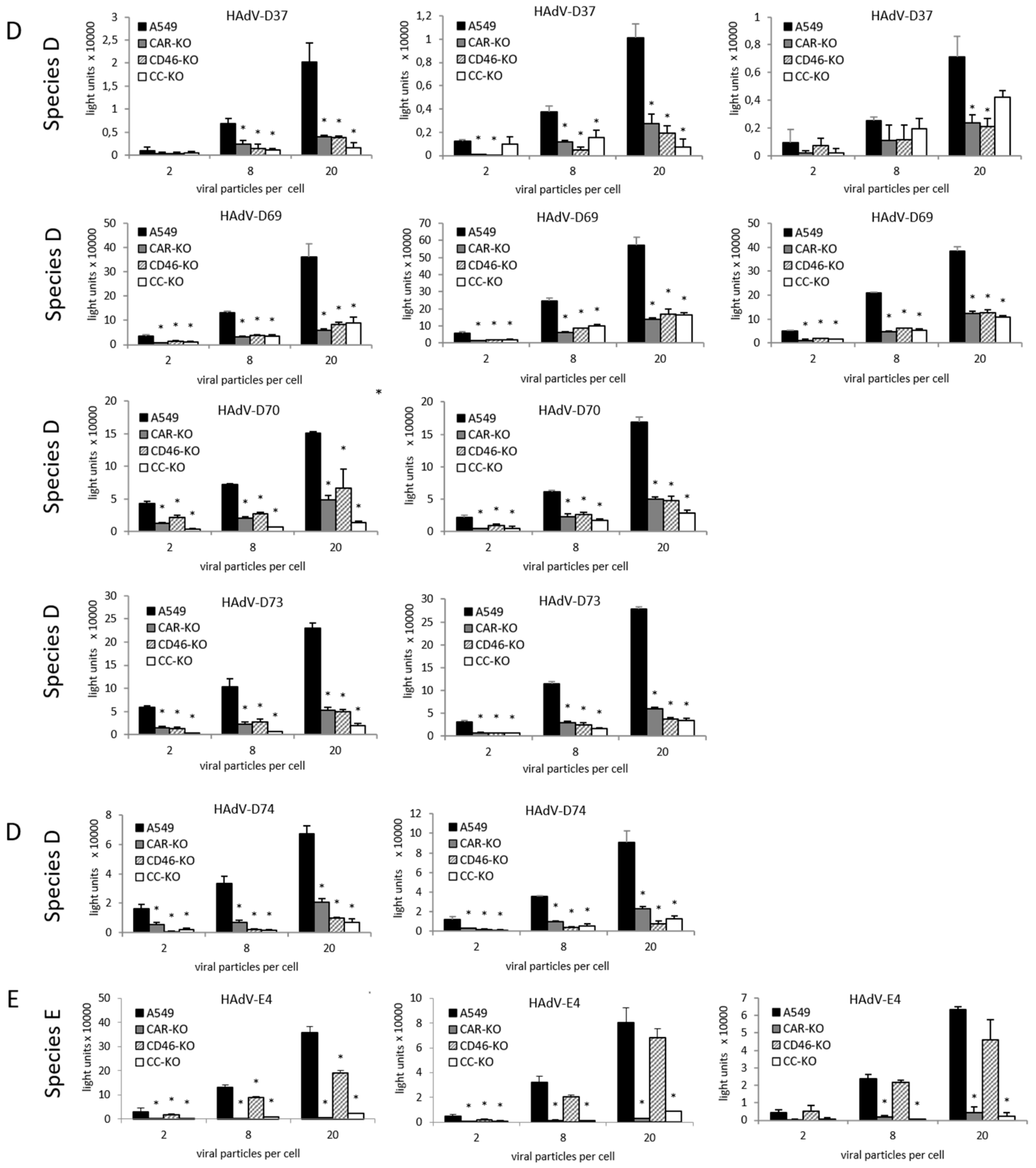

Figure S4. Screening of receptor usage in adenovirus species B to E, independent expreiments. Parental A549-, CAR-KO-, CD46-KO-, CC-KO-cells were transduced with respective viruses at various viral particle numbers per cell (VP/C: 2, 8, 20). Luminescence levels were analyzed 26 hours post infection by an ELISA plate reader. Uninfected cells were used to set the background level. Each 96-well-plate was seeded with 30 x 10^3 cells. Infection was carried out 4 hours after cell seeding. Each bar represents the mean of triplicated wells (96-well plate). Error bars represent standartdeviation. Each of the KO-cell lines was compared to the A549 cell line value. Significant luminescence reductions are marked by *(p<0,05). Each graph represents data from one out of three independent experiments. D, The CAR-KO signal in the third experiment exceeds the signal in A549. This is most probably due to unprecise pipetting with falsely high virus concentrations in the CAR-KO samples.
